# Quanzhenyiqitang Reverses LPS-Induced Inflammation via Inhibiting PYK2/p38MAPK/HDAC2/CK2 Signaling Pathway in Rat Alveolar Macrophage

**DOI:** 10.1155/2022/7857022

**Published:** 2022-01-10

**Authors:** Ke-Qiang Chen, Da-zhi Li, Zhi-bin Chen, Chuan-lin Zhang, Bin-can Wang, Chun-e Wang

**Affiliations:** Department of Respiratory and Critical Care Medicine, The Second Affiliated Hospital of Fujian Traditional Chinese Medical University, Fuzhou, China

## Abstract

Chronic obstructive pulmonary disease (COPD) is a common chronic pulmonary disease with multiple etiologies and pathological changes. PYK2 expression is significantly increased in lipopolysaccharide-induced lung injury, but it mediates chronic lung inflammation. The mechanism of its occurrence remains unclear. Quanzhenyiqitang is often used in clinical treatment of COPD, so this study explored the mechanism of its treatment of lipopolysaccharide-induced lung injury. In this study, transfection, flow cytometry, QRT-PCR, and Western blotting methods were used to study the mechanism of Quanzhenyiqitang lipopolysaccharide-induced lung injury. The results showed that the mechanism of occurrence remains unclear. Our novel observations imply that the PYK2/p38MAPK/HDAC2/CK2 pathway is one of the fundamental underlying mechanisms that mediate the pathogenic progression of COPD, and Quanzhenyiqitang may be the therapeutic drug to prevent chronic inflammation and delay the progression of COPD by inhibiting PYK2 signaling pathways.

## 1. Introduction

Macrophages are an important component of the human body's innate immunity. It was originated in the yolk sac and liver precursor cells during embryonic development and then entered into the lungs and settled to form alveolar macrophages [1]. Alveolar macrophages are mostly distributed in the alveolar cavity, accounting for 80% of the alveolar resident cells. It is the only inner cell group that contacts with air in the human body in the first line of defense against pollutants and pathogenic bacteria [2]. It can secrete more than one hundred types of inflammatory mediators. These substances further trigger an innate immune response in the normal lung tissues. According to the activation pathway, alveolar macrophages have two phenotypes: classic activated macrophages (M1 macrophages) activated classically and alternately activated macrophages (M2 macrophages) activated in a selective pathway [3]. During the inflammatory response, M1 and M2 are involved in mediating the innate immune response and various inflammatory responses. M1 macrophages respond to microbial factors and Th1 proinflammatory cytokines, exhibit glycolysis, and are associated with the release of inflammatory cytokine, enhancing bacterial killing, and recruitment of immune cell into the lung parenchyma and alveoli [4]. In contrast, M2 macrophages exposed to Th2 cytokines induce the oxidative metabolism, which is related to the release of anti-inflammatory cytokines, phagocytosis (apoptosis) of apoptotic cells, and collagen deposition, thus contributing to the resolution and damage of inflammation [5]. Being highly plastic and highly responsive to environmental signals, alveolar macrophages can rapidly mediate inflammatory phenotypes, and the changes of alveolar macrophages are reversible. In the process of lung inflammation, macrophages continue to interact with epithelial cells, microvascular endothelial cells, neutrophils, lymphocytes, fibroblasts, and stem cells or tissue progenitor cells to regulate the stability of the lung environment and the pathogens [5]. The polarized states of alveolar macrophages are not mutually exclusive, and cells can simultaneously display two subtypes of M1 and M2 macrophages according to environmental signals.

There have been a series of published articles, reviews, and research reports that emphasize the role of alveolar macrophages in lung inflammation. Feller et al. found that continuous smoking activated the atypical Wnt5a-PPAR*γ* signaling, leading to macrophage polarization from M2 to M1, lung inflammation, and eventually chronic obstructive pulmonary disease [6]. In rats exposed to endotoxin, alveolar macrophages, and interleukin-6, toll-like receptor 4 was significantly upregulated in vivo. Lee et al. discovered that alveolar macrophages interact with lung epithelial cells via extracellular vesicles and intracellular vesicles [7]. Through extracellular vesicle-mediated signal transduction, the bidirectional paracrine interaction between macrophages and epithelial cells may trigger an inflammatory cascade in the lung. In a mouse model of bleomycin-induced pulmonary fibrosis, Elewa et al. reported an increase in the CD80^+^ M1 macrophage subpopulations, and the number of macrophage infiltration in the lung and mediastinal fat-related lymphoid clusters was significantly positively correlated [8]. This study suggests that macrophage infiltration of the lung and mediastinal fat-associated lymphoid clusters may be profoundly involved in the progression of pulmonary inflammatory diseases.

As a nonreceptor protein tyrosine kinase, proline-rich tyrosine kinase-2 (PYK2) can sense a variety of extracellular signaling molecules and participate in intracellular signaling pathway transmission. The activation of its downstream pathway plays an important role in the pathogenesis of airway stenosis and fibrosis, lung parenchyma destruction, and mucus hypersecretion in chronic lung inflammation [9]. PYK2 and its downstream pathways play a crucial role in the pathogenesis of airway stenosis and fibrosis, lung parenchyma destruction, and mucus hypersecretion in chronic lung inflammation. At present, there are few studies concerning the downstream mechanism of PYK2. It has not been shown how the PYK2 signal transduction pathway could be involved in the pulmonary inflammatory response associated with alveolar macrophages. We speculate that the PYK2/p38MAPK/HDAC2/CK2 pathway might be one of the fundamental mechanisms of LPS-induced lung injury and is the key to the development of new strategies for the prevention and treatment of chronic lung inflammatory diseases.

To scrutinize the role of the PYK2-mediated p38MAPK/HDAC2/CK2 signaling pathway in LPS-induced inflammation of alveolar macrophages, we directly investigated the expression levels of PYK2 and downstream signaling pathways in alveolar macrophages stimulated by LPS. In rat alveolar macrophages stimulated by LPS, PYK2 was phosphorylated, resulting in changes in the downstream p38MAPK/HDAC2/CK2 signaling pathway, which ultimately affects the function and inflammation of alveolar macrophages. Quanzhenyiqitang is widely used in the treatment of various senile diseases with deficiency, such as chronic heart failure, chronic obstructive pulmonary disease, and lung cancer [10]. Targeting PYK2 by lentiviral transfection technology or Quanzhenyiqitang-treated serum, we found that the PYK2/p38MAPK/HDAC2/CK2 signaling pathway significantly contributed to the inflammatory response of alveolar macrophages induced by LPS stimulation. In conclusion, our results indicate that PYK2 mediates the p38MAPK/HDAC2/CK2 signaling pathway in LPS-induced alveolar macrophage inflammation and Quanzhenyiqitang may be the potential drug that could reverse this immune response.

## 2. Materials and Methods

### 2.1. Cell Culture

Rat alveolar macrophages NR8383 were obtained from the Cell Bank of the Chinese Academy of Sciences (Shanghai, China) and contained 10% fetal bovine serum, 100 U/mL penicillin, and 100 mg/L streptomyces in a humidified incubator at 37°C and 5% CO_2_ DMEM medium.

### 2.2. Acquisition of Quanzhenyiqitang-Treated Serum Acquisition

Sixty SPF male SD rats were 4–6 weeks old and weighed 180–220 g, drink and eat freely for 7 days to acclimate to the environment. 15 g ginseng, 15 g red peony, 15 g *Rehmannia glutinosa*, 6 g aconite, 6 g *Atractylodes*, 15 g *Achyranthes*, and 6 g *Schisandra* had been decocted for 20 minutes. Each rat was given by gavage twice a day, 5 ml each time with Quanzhenyiqitang, for 7 days. On day 7, after 12 hours of fasting, the rats were gavaged twice with a complete daily dose [10]. For Quanzhenyiqitang-untreated group, each rat was lavaged with 5 ml saline solution at the same time. One hour after the administration, the rats were anesthetized by intraperitoneal injection of pentobarbital sodium (0.2 ml/100 g). After routine sterilization, we collect abdominal aortic blood (approximately 6 ml) and transfer it into a negative pressure container using a puncture needle under sterile conditions. The blood sample was then placed in a 37°C waterbath for 15 minutes and centrifuged at 400x g for 15 minutes. The serum was filtered through a 0.22 *μ*m filter and transferred into new Eppendorf tubes and stored at −20°C.

### 2.3. Design and Transfection of Pyk2 siRNAs

siRNAs were obtained from RiboBio and transfected according to the manufactures' instructions. To put it simply, siRNA was diluted with 200 *µ*L Opti-MEM. After gently inverting and mixing the transfection reagents, 4 *µ*L Lipofectamine^TM^ 2000 was also diluted with 200 *µ*L Opti-MEM. Transfection reagent and siRNA diluent were then mixed and placed in a 6-well cell plate. siRNA sequences are given in Table 1.

### 2.4. Apoptosis Assay

After performing corresponding stimulation, cells were centrifuged at 1000 g for 5 minutes. The supernatant was discarded, and the cells were then resuspended gently with PBS and counted. After adding Annexin V-FITC binding solution and propidium iodide staining solution, the cells were incubated at room temperature (20–25°C) in the dark for 10–20 minutes and then transferred onto the board for testing.

### 2.5. ELISA Detection

After cell supernatants were centrifuged and collected, a series of performances including antigen coating, primary antibody reaction, secondary antibody binding, substrate preparation, and microplate reader were completed according to the manufacturer's instructions. The process was repeated three times for all samples.

### 2.6. Real-Time Quantitative PCR

RNA samples were extracted with quality tests meeting the onboard standards. The extended reverse transcription products were amplified and quantified by the SYBR Green master mixes detection kit. Primer sequences are given in Table 2.

### 2.7. Western Blotting

Cell samples were washed twice with PBS, lysed with lysis buffer for 10–15 min, and then sonicated in 200 W for 4 times, 5 sec each time, at 2 s intervals. Being centrifuged in 12000x g for 15 min, the protein concentrations of each sample were determined by the BCA method. Following sample loading, transferring, milk blocking, primary antibody incubation, and secondary antibody incubation, the exposed band showed the expression of the protein of interest. The detailed information of antibodies is given in Table 3.

### 2.8. Statistical Analysis

Statistical analysis was performed using SPSS17.0. Student's *t*-test was employed to compare the result between two groups, and one-way ANOVA was used to assess the difference among more than two groups. The data are presented in the form of mean ± SEM. *P* value <0.05 was considered significant.

## 3. Results

### 3.1. Quanzhenyiqitang-Treated Serum Inhibits LPS-Stimulated PYK2 Signaling Pathways in Rat NR8383 Cell

**Figure 1:**
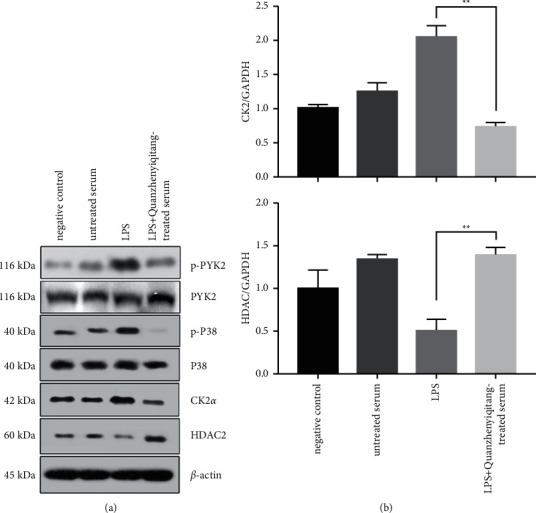
Expression of p-Pyk2, p-p38MAPK, HDAC2, and CK2 after LPS induction. (a) The gel figure of p-Pyk2, p-p38MAPK, HDAC2, and CK2 after LPS induction. (b) The expression of p-Pyk2, p-p38MAPK, HDAC2, and CK2 after LPS induction.

Western blotting and qRT-PCR were performed to scrutinize the effect of Quanzhenyiqitang-treated serum on the LPS-stimulated Pyk2/p38MAPK/HDAC2/CK2 signaling pathway in rat alveolar macrophage. The results showed that the expression of p-Pyk2, p-p38MAPK, and CK2 was activated, while HDAC2 was downregulated after LPS stimulation of rat alveolar macrophages, and Quanzhenyiqitang-treated serum significantly inhibited this progression (Figure 1(a)). The same result was also obtained in the expression levels of HDAC2 and CK2 mRNA (Figure 1(b)).

### 3.2. Verification of Pyk2 siRNA Interference Efficiency

**Figure 2:**
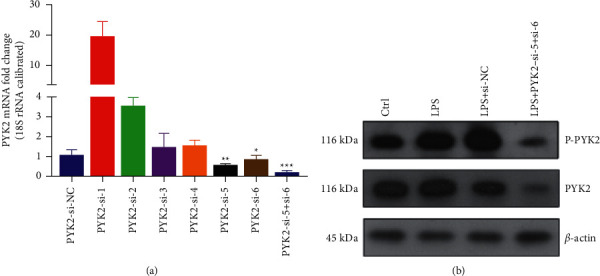
Verification of Pyk2 siRNA interference efficiency by qRT-PCR and Western blotting. (a) The verification of Pyk2 siRNA interference efficiency by qRT-PCR. (b) The verification of Pyk2 siRNA interference efficiency by Western blotting.

To verify the potential role of Pyk2 in macrophage, we designed six Pyk2 siRNA fragments, and by the method of qRT-PCR, found that siRNA-5 and siRNA-6 had a better effect than others, but the efficiency was still not satisfactory (Figure 2(a)). So, we chose a combination of siRNA-5 and siRNA-6 to treat rat alveolar macrophage. The qRT-PCR result showed that the inhibitory rate was up to 80%. Western blot also showed that the expression levels of Pyk2 and p-Pyk2 were both significantly decreased (Figure 2(b)).

### 3.3. Pyk2 siRNA Silencing or Quanzhenyiqitang-Treated Serum Enhancing the Function of Rat Alveolar Macrophage

**Figure 3:**
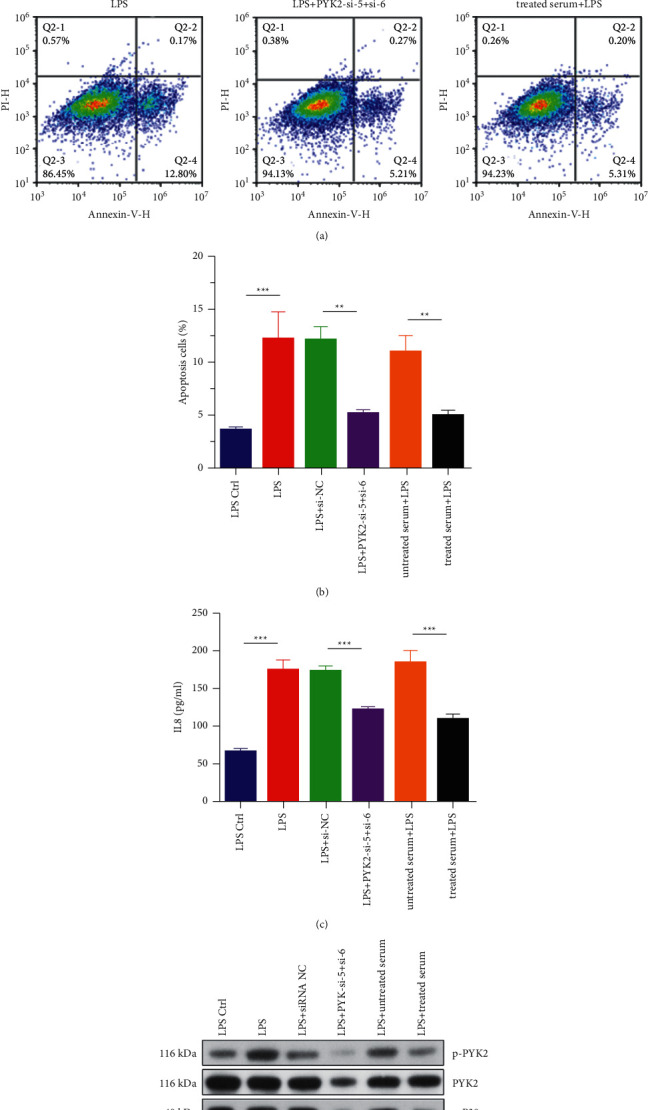
The function of the rat alveolar macrophage and signaling pathway after Pyk2 siRNA silencing or Quanzhenyiqitang-treated serum stimulation. (a) Cell apoptosis detected by flow cytometry after Pyk2 siRNA silencing or Quanzhenyiqitang-treated serum stimulation. (b) Apoptosis rate after Pyk2 siRNA silencing or Quanzhenyiqitang-treated serum stimulation. (c) IL-8 level after Pyk2 siRNA silencing or Quanzhenyiqitang-treated serum stimulation. (d) The gel figure of proteins after Pyk2 siRNA silencing or Quanzhenyiqitang-treated serum stimulation.

After LPS stimulation, the apoptosis rate of rat alveolar macrophage NR8383 cell was significantly increased, compared with the LPS Ctrl group (Figure 3(a)). While, cotransfection of rat alveolar macrophages with Pyk2 siRNA-5 and Pyk2 siRNA-6 or Quanzhenyiqitang-treated serum alleviates the apoptosis effect significantly (Figure 3(b)). ELISA results showed that after LPS stimulation, the secretion of inflammatory mediator IL-8 in rat alveolar macrophage was significantly increased. However, after Pyk2 siRNA transfection or Quanzhenyiqitang-treated serum stimulation, IL-8 secretion in rat alveolar macrophage was inhibited to a certain extent. The results indicate that targeting Pyk2 expression could inhibit alveolar macrophages from producing inflammatory mediator IL-8 (Figure 3(c)). After LPS stimulated rat alveolar macrophages, the expression of p-Pyk2, p-p38MAPK, and CK2 upregulated, while HDAC expression was downregulated significantly, and the expression trends of HDAC2 and CK2 mRNA levels were also similar to the previous result. Nevertheless, after Pyk2 siRNA transfection or Quanzhenyiqitang-treated serum stimulation, this process was blocked (Figure 3(d)).

### 3.4. Quanzhenyiqitang-Treated Serum Alleviates the Side Effect of Pyk2 OE on Rat Alveolar Macrophage

**Figure 4:**
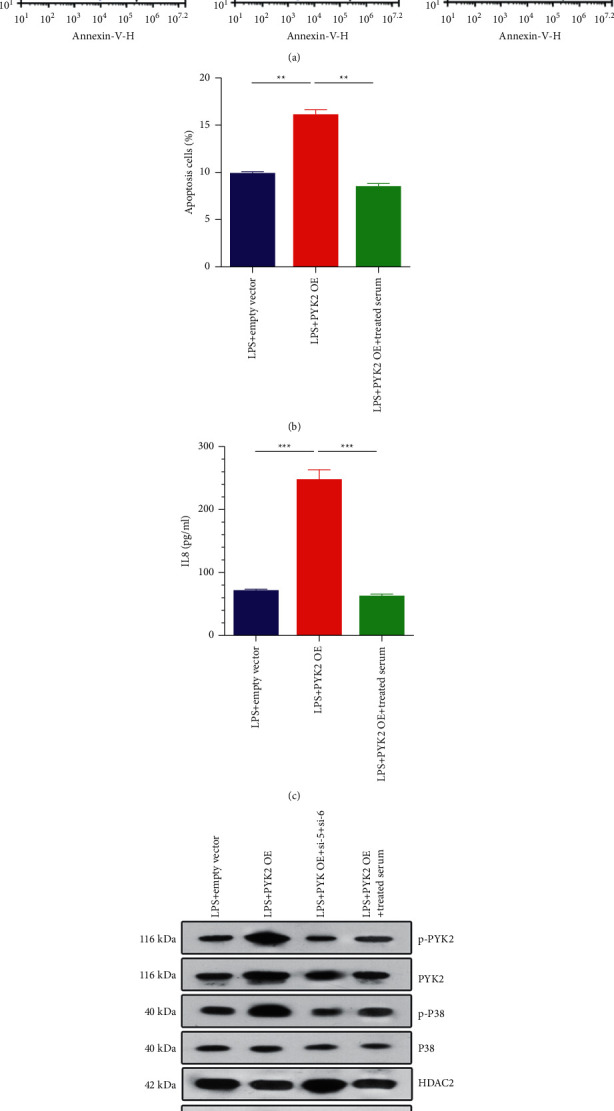
Quanzhenyiqitang-treated serum alleviates the side effect of Pyk2 OE on rat alveolar macrophage (a) Cell apoptosis detected by flow cytometry after Quanzhenyiqitang-treated serum alleviates the side effect of Pyk2 OE on rat alveolar macrophage. (b) Apoptosis rate after Quanzhenyiqitang-treated serum alleviates the side effect of Pyk2 OE on rat alveolar macrophage. (c) IL-8 level after Quanzhenyiqitang-treated serum alleviates the side effect of Pyk2 OE on rat alveolar macrophage. (d) The gel figure of proteins after Quanzhenyiqitang-treated serum alleviates the side effect of Pyk2 OE on rat alveolar macrophage.

After cotransfection of rat alveolar macrophages with Pyk2 OE, the apoptosis process was exacerbated significantly (Figure 4(a)). However, after Quanzhenyiqitang-treated serum stimulation, the apoptosis rate was decreased significantly compared with the Pyk2 OE group (Figure 4(b)); ELISA results also showed that after Quanzhenyiqitang-treated serum stimulation, the secretion level of inflammatory mediator IL-8 in rat alveolar macrophage was downregulated significantly compared with the Pyk2 OE group. Meanwhile, after Pyk2 siRNA transfection or Quanzhenyiqitang-treated serum stimulation, IL-8 secretion in rat alveolar macrophage was inhibited to a certain extent. This indicates that targeting Pyk2 expression could inhibit alveolar macrophages from producing inflammatory mediator IL-8 (Figure 4(c)). Western blot results also showed that after Pyk2 OE transfection, the expression of p-Pyk2, p-p38MAPK, and CK2 upregulated while HDAC2 was downregulated, as well as the expression levels of HDAC2 and CK2 mRNA levels and Quanzhenyiqitang-treated serum stimulation inhibited this signaling pathway cascade (Figure 4(d)).

## 4. Discussion

In this study, we investigated the role of the Pyk2-mediated p38MAPK/HDAC2/CK2 signaling pathway in LPS-induced inflammation of rat alveolar macrophages and focused on the effect of potential therapeutic drug Quanzhenyiqitang on this signaling pathway. The results showed that LPS could induce apoptosis of alveolar macrophages. In addition, LPS stimulated the phosphorylation of Pyk2 in rat alveolar macrophages through LPS, thus activating downstream factors p38MAPK, HDAC2, and CK2, which in return induced the secretion of the inflammatory mediator IL-8. We also found that transfection of alveolar macrophages with Pyk2 siRNA-5/-6 or Quanzhenyiqitang-treated serum stimulation could effectively inhibit the LPS-induced apoptosis process of rat alveolar macrophages and decrease the secretion of the inflammatory mediator IL-8.

Alveolar macrophages are known as the first line of defense for the lungs against pathogenic microorganisms and lung damage. They are the dominant inflammatory cells in the lungs and can be directly activated by tobacco extracts to release inflammatory regulators [10]. The number of alveolar macrophages is increased in chronic inflammation environments such as COPD airway, lung, and bronchoalveolar lavage fluid, and sputum of patients with sexual lung disease increased. Previous studies have shown that LPS pretreatment can accelerate the inflammatory response and mimic the pathological process of COPD [11]. It is a prevalent method to create a COPD-like mouse model within a short exposure period and expose mice to CS and LPS for 3 consecutive months [12]. Therefore, this study used LPS to induce an inflammatory response in rat alveolar macrophages and to explore the effects and potential mechanisms of Pyk2 in LPS-stimulated inflammatory response. We know that IL-8 was synthesized and secreted by monocytes, macrophages, endothelial cells, fibroblasts, and epidermal cells under the activation of stimulating factors [13]. As far as known, IL-8 was the strongest neutrophil-activating factor and chemokine, which can effectively dilate blood vessels and promote the proliferation of blood vessels. Neutrophils are the target cells for the activation of IL-8 [14]. IL-8 can specifically chemoattract neutrophils into inflammatory tissues, promoting their degranulation and producing superoxide anions, finally causing respiratory outbreaks, and activating inflammatory cells. Aband AR et al. found in vitro experiments that inhibiting the activity of Pyk2 by transfection in endothelial cells can prevent the process of LPS-induced IL-8 production [15]. Our results indicated that LPS could cause apoptosis of rat alveolar macrophages and stimulated cells to secrete IL-8 inflammatory mediators. These results demonstrated that LPS indeed induced cellular inflammation. Interestingly, we observed that after LPS-induced alveolar macrophages, apoptosis and IL-8 secretion levels were significantly reduced after Pyk2 siRNA-5 and Pyk2 siRNA-6 transfection or Quanzhenyiqitang-treated serum stimulation. Therefore, our study novelly confirmed that this inhibition of Pyk2 phosphorylation alleviated the apoptosis of alveolar macrophages and the release of inflammatory mediator IL-8.

Proline-rich tyrosine kinase (proline-rich tyrosine kinase-2, Pyk2) participates in intracellular signaling pathways, regulates cell movement, regulates cell proliferation and migration, apoptosis, and other processes. Previous studies have shown that Pyk2 regulated the chemotaxis of macrophages [16]. In Pyk2-knockout mice, the capability of stimulating factors to promote macrophage infiltration into tissues was significantly reduced [17]. The latest research showed that Pyk2 played an important role in acute lung injury. Duan Y et al. confirmed that inhibition of Pyk2 protein in serious LPS-induced acute lung injury mice was the key to inhibiting acute lung inflammation and edema formation [18]. In macrophages chemoattracted by LPS, Pyk2 is also closely associated with the secretion of cytokines. Under the condition that a chemical inducer was added to the lung tissue, as long as the activity of Pyk2 was inhibited, the process of LPS-induced neutrophil exudation could also be blocked. The results of our study showed that LPS induced a high expression of Pyk2 and p-Pyk2 in rat alveolar macrophages. At the same time, we determined the impact of LPS on p38 MAPK and CK2 in alveolar macrophages. Pyk2-mediated p38MAPK/HDAC2/CK2 was the key to LPS-induced inflammation of alveolar macrophages. We conducted Pyk2 functional experiments using Pyk2 siRNA transfection and Quanzhenyiqitang-treated serum stimulation.

Protein kinase CK2, also known as casein kinase-2 (casein kinase II), is a highly conserved second messenger-independent protein kinase that plays a crucial role in cell growth, proliferation, and apoptosis. Therefore, CK2 has become a promising therapeutic target. CK2a expression and activity levels increase in many inflammatory pathologies [19]. Many proteins involved in inflammatory signaling pathways can interact with CK2a, such as NF-*κ*B, CREB, CREM, c-Jun, c-Fos, c-Myc, and Max. The p38MAPK inhibition by applying the p38MAPK inhibitor SB203580 also inhibited the activation of CK2a [20], which implied that CK2a was downstream of the p38MAPK pathway. Our results showed that CK2 was highly expressed in LPS-stimulated rat alveolar macrophages, and its trend was consistent with Pyk2. Inhibiting Pyk2 expression by Pyk2 siRNA transfection and Quanzhenyiqitang-treated serum stimulation inhibited Pyk2 expression; the phosphorylation level of p38MAPK was also consistent with Pyk2 which verified that CK2 was the downstream factor of Pyk2 and p38MAPK.

Histone deacetylase (HDAC) is the key component in the modification of chromosome structure and the regulation of gene expression. In the nucleus, the process of histone acetylation and histone deacetylation is in a dynamic balance and is jointly regulated by HDAC [21]. The function of HDAC is to transfer the acetyl group on the lysine residue of the chromosomal core histone, resulting in the condensation of DNA, and reducing the binding of transcription factors to their binding sites, thereby inhibiting the transcription of genes. HDAC2 is the major histone deacetylase subtype involved in the pathogenesis of COPD. The phosphorylation level of HDAC2 depends on the level of CK2a protein kinase. During the phosphorylation of HDAC2, it is mainly regulated by CK2a. Being knocked out of the CK2a gene can effectively reduce the phosphorylation of HDAC2 [22]. CK2a can promote the phosphorylation of HDAC2, thereby losing the ability to acetylate. The activity and expression of HDAC2 in the alveolar cells, airways, and lung parenchyma of COPD patients and normal smokers are reduced, thereby activating the expression of IL-8 and other inflammatory factors. Studies have shown that in patients with COPD, with the increase of the inflammation indicators, C-reactive protein (CRP) and procalcitonin (PCT) increase, and HDAC2 levels gradually decrease. In COPD patients treated with hormones, the expression of HDAC2 was upregulated, and NF-*κ*B inhibited, leading to inhibition of the expression of IL-8 and other inflammatory factors, and finally, the patient's airway inflammation was alleviated. Our research showed that HDAC2 was highly expressed in LPS-stimulated rat alveolar macrophages, and Pyk2 siRNA transfection and Quanzhenyiqitang-treated serum stimulation inhibited Pyk2 expression and downregulated the expression of HDAC2 which indicated that HDAC2 is profoundly involved and played an important role in the inflammatory response mediated by Pyk2 in alveolar macrophages.

## 5. Conclusion

In summary, we have demonstrated that LPS induced the apoptosis process of rat alveolar macrophages and secrete the inflammatory mediator IL-8 by upregulating the levels of Pyk2 and downstream factors p38MAPK, HDAC2, and CK2. Pyk2 siRNA transfection and Quanzhenyiqitang-treated serum stimulation could reverse these trends, which may provide a new strategy for the treatment of COPD patients.

## Figures and Tables

**Table 1:** siRNA sequences for rPtk2b-siRNA.

rPtk2b-siRNA-1 sense	GCUAUUUGCCAGAAGACUUTT
rPtk2b-siRNA-1 antisense	AAGUCUUCUGGCAAAUAGCGG
rPtk2b-siRNA-2 sense	GCAUAGAGUCAGACAUCUATT
rPtk2b-siRNA-2 antisense	UAGAUGUCUGACUCUAUGCTA
rPtk2b-siRNA-3 sense	CCGUGAAGAUGUAGUUCUUTT
rPtk2b-siRNA-3 antisense	AAGAACUACAUCUUCACGGGC
rPtk2b-siRNA-4 sense	GCAGCCUUUCUUCUGGCUUTT
rPtk2b-siRNA-4 antisense	AAGCCAGAAGAAAGGCUGCTT
rPtk2b-siRNA-5 sense	GCAGUCUCAGUGACAUUUATT
rPtk2b-siRNA-5 antisense	UAAAUGUCACUGAGACUGCAC
rPtk2b-siRNA-6 sense	GCUUGGACCCGAUGGUUUATT
rPtk2b-siRNA-6 antisense	UAAACCAUCGGGUCCAAGCAT

**Table 2:** Primer sequences.

Name	Sequence (5'-3')
rPtk2b-qF	CTGGAGAGCATCAACTGTGTGC
rPtk2b-qR	GATGGGTAGACGTGTCACAGAG
rHDAC-qF	CAACCTAACTGTCAAAGGTCACGC
rHDAC-qR	TGAAGTCTGGTCCAAAATACTCGA
rGAPDH-qF	TGATTCTACCCACGGCAAGTT
rGAPDH-qR	TGATGGGTTTCCCATTGATGA

**Table 3:** The detailed information of antibodies.

Actin	PYK2	p-PYK2	P38	p-P38	CK2	HDAC2

Abcam	Abcam	Abcam	Abcam	Abcam	R&D	Abcam
ab179467	ab32571	ab4800	ab170099	ab4822	MAB7957	ab32117
1 : 3000	1 : 2000	1 : 1000	1 : 3000	1 : 1000	1 : 2000	1 : 2000

## Data Availability

The data used to support the findings of this study are available from the corresponding author upon request.
